# Evaluation of the impact of body mass index on venous thromboembolism risk factors

**DOI:** 10.1371/journal.pone.0235007

**Published:** 2020-07-09

**Authors:** Fatemeh Tajik, Mingzheng Wang, Xiaohui Zhang, Jie Han

**Affiliations:** 1 School of Economics and Management, Dalian University of Technology, Dalian, China; 2 School of Management, Zhejiang University, Hangzhou, China; 3 Business School, University of Exeter, Exeter, England, United Kingdom; 4 The First Affiliated Hospital, Zhejiang University, Hangzhou, China; University of Murcia, SPAIN

## Abstract

In this paper, we investigate the interaction impacts of body mass index (BMI) on the other important risk factors for venous thromboembolism (VTE), using deep venous thrombosis (DVT) patient data from the International Warfarin Pharmacogenetics Consortium (IWPC). We apply eight machine learning techniques, including naive Bayes classifier (NB), support vector machine (SVM), elastic net regression (ENET), logistic regression (LR), lasso regression (LAR), multivariate adaptive regression splines (MARS), boosted regression tree (BRT) and random forest model (RF). The RF method is selected as the best model for classification. Out of 33 features considered in this study, we identify 12 variables as relatively important risk factors for VTE. Finally, we examine the interaction impacts of BMI on these important VTE risk factors. We conclude that the impacts of risk factors on VTE incidence are varying across different BMI groups, and the variations are different for different risk factors. Therefore the interaction impacts of BMI on the other risk factors have to be taken into account in order to better understand the incidence of VTE.

## 1 Introduction

Venous thromboembolism (VTE), a term referring to blood clots in the veins, is a disorder that includes deep vein thrombosis (DVT) and pulmonary embolism (PE). It is the third most common vascular disorder in Caucasian after myocardial infarction and stroke [1, 2]. VTE also causes morbidity and mortality in cancer patients and patients after major surgery, especially hip or knee arthroplasty [3–5]. To prevent VTE, warfarin is one of the main oral anticoagulants treatments [6, 7], which needs international normalized ratio (INR) monitoring.

One leading risk factor for VTE is obesity [1, 3, 8, 9]. In particular, Yang et al. [10] classified obesity as a moderate risk factor for VTE incidence, and found it can interact with other risk factors in VTE development and increase the risk of VTE. Obesity not only increases the VTE incidence, but also causes other chronic diseases such as diabetes, hypertension, coronary heart disease, and ischemic stroke [10]. The prevalence of obesity has increased dramatically during the last decades [11, 12]. According to the World Health Organization, at least 700 million adults and more than 20% of young children aged 6–11 years in the world were obese in 2015 [13]. Due to the high prevalence of obesity, there is increasing interest in measuring body fat. Body mass index (BMI), calculated as the ratio of an individual’s weight (in kg) to his/her squared height (in meter), is one of the most popular measures of body mass because of its simplicity.

BMI, as an indicator for obesity, has been identified as a VTE risk factors by the previous studies [14–18]. The previous studies have also found the effect of BMI on warfarin treatment. For example, Routledge et al. [19] identified one of the key factors affecting on warfarin therapy is body weight. Tellor [20] showed morbidly obese patients required higher total weekly dose to maintain a therapeutic INR. Wallace et al. [21] compared average warfarin dose with the different patients BMI to get the therapeutic INR in hospital, and found warfarin response dose was decreasing with the BMI increment. Wells et al. [22] predicted warfarin dose with BMI, age and some other predictors, for patients with a history of VTE. However, there is no enough evidence on the impact of BMI on VTE occurrence for warfarin treated patients with history of DVT, and on the interaction between BMI and other risk factors for VTE.

To assess the VTE risk factors, the traditional statistical techniques such as linear regression model [17, 22], logistic regression model [5] and Cox regression model [23] have been widely used by the previous studies. Although these statistical techniques are powerful tools for prediction and description, there are various issues influencing classification, i.e. prediction of discrete values, by the traditional regression models [24, 25]. In particular, highly correlated predictors and sparse sample size are two common statistical issues which may lead to collinearity and over-fitting, respectively [26]. To address these issues, machine learning (ML) techniques provide an alternative way. Significant advantages of ML techniques include high power and accuracy, ability of modeling non-linear effects, and capacity of dealing with large data sets [27, 28].

The objective of ML algorithms is to optimize its performance at a particular task using the past experience (input data). Supervised classification techniques are ML algorithms that learn patterns in data to predict associated discrete classes [29]. In medical science, supervised classification techniques have been employed to identify risk factors for a specific disease or to predict disease occurrence such as VTE. Among a large number of available supervised ML techniques, kernel machine learning [30], various decision trees [31, 32], artificial neural networks [33–35], random forest [36, 37], support vector machines [38, 39], Bayesian decision rules [40, 41], supervised principal component analysis [42], penalized regression models [43] have been applied in medical science. Although the choice of ML techniques is often based on the minimum loss function, it is difficult to make an informed decision on the most appropriate method.

The main objective of this study is to investigate the interaction effect of BMI on the other risk factors for VTE. In order to fulfill this objective, we have answered the following two questions first. They are, (1) which ML algorithm is most suitable for classification, and (2) which risk factors play important roles as risk factors for VTE. In particular, we use the DVT patient’s validation group of international warfarin pharmacogenetics Consortium (IWPC), from the PharmGKB website to identify the risk factors for VTE, predict the VTE occurrence, and examine the interaction impact of BMI on the other risk factors for VTE. We perform a two-step procedure to select important risk factors for VTE. First, we apply eight ML methods, including naive Bayes classifier (NB), support vector machine (SVM), elastic net regression (ENET), logistic regression model (LR), lasso regression (LAR), multivariate adaptive regression splines (MARS), boosted regression tree (BRT) and random forest classification (RF), and select the best classification method among them. Second, we use the selected classification model to identify the important risk factors for VTE. Finally, we examine the interaction impacts of BMI on the selected risk factors for VTE.

## 2 Methods

In this study, we apply eight supervised classification ML methods. We compare their performance and select the best model to identify the important risk factors for VTE. A brief introduction of each ML technique is given below.

### 2.1 Naïve Bayesian classifier

Naïve Bayesian classifier (NB) is a simple probabilistic classifier based on the Bayes’ theorem [29], which is introduced by Maron [44]. NB assumption is conditional independence between every pair of predictors [45]. It predicts membership probabilities for each class, such as the probability that a given record or a data point belongs to a particular class. The class with the highest probability is considered as the most likely class, which is also known as maximum posterior probability of each class [46].

### 2.2 Support Vector Machine (SVM)

Introduced by Vapnik [47], Support Vector Machine (SVM) is based on the inductive learning [48]. SVM is used in both classification and regression. It uses the principle of maximum margin classifier to separate data. For a d-dimensional data, SVM uses a (d– 1)-dimensional hyper plane for data separation. The advantages of SVM are in identifying nonlinear impact using small sample and high dimensional data [49]. In addition, its loss function is based on a global optimization, hence SVM is not prone to fall into a local optimization [50].

### 2.3 Elastic-net regression

Elastic-net Regression (ENET) was introduced by Zou [51], which combines the lasso regression (LAR) and the ridge regression model. It penalizes both the L_1_ and L_2_ norms with individual tuning parameters in order to achieve the best performance for both LAR and ridge regressions. ENET is robust to extreme correlations among the predictors [52].

### 2.4 Logistic regression model

Pearl et al. [53] introduced the logistic regression model (LR), based on logistic function, to model a binary dependent variable. It has been borrowed by machine learning for binary classification problems.

### 2.5 Lasso regression

Lasso (Least absolute shrinkage and selection operator) regression (LAR) analysis performs both variable selection and regularization. It was introduced by Tibshirani [54] in order to improve the prediction accuracy of regression model by selecting only a subset of the provided covariates for use in the final model rather than using all of them. LAR relies on the L_1_ penalty for both fitting and penalization of the coefficients.

### 2.6 Multivariate adaptive regression splines

Multivariate adaptive regression splines (MARS) is a non-parametric regression technique introduced by Friedman [55]. The MARS uses spline-based method by allowing different functions (linear or nonlinear) over different intervals to model the nonlinear relationship between the input and the output variables (x, y), in order to improve the goodness of fit [56].

### 2.7 Boosted regression tree model

Boosted Regression Tree (BRT) models combine two techniques, i.e. decision tree algorithms and boosting methods [57]. In particular, a decision tree algorithm relates a response to their predictors by recursive binary splits, and a boosting method is an adaptive method for combining many simple models to give improved predictive performance.

### 2.8 Random forests classifier

Random Forests (RF) classifier is an ensemble tree-based learning algorithm [58]. Generally speaking, RF constructs a multitude of decision trees at training time and outputs the class as the mode of the classes (classification) of the individual trees. The first algorithm for random forests was created by Ho [59], and the random forests proper was first introduced by Breiman [60]. RF is robust to overfitting, and more stable in the presence of outliers and in high dimensional parameter spaces than other machine learning algorithms [61].

### 2.9 Simulation setup

Feature (or variable) selection helps to explain the data in the simplest way, avoids unnecessary cost of measuring redundant predictors, and mitigate the issue of collinearity. It also helps to improve the prediction accuracy [62]. Therefore finding a proper feature selection method is crucial. In this paper, we apply the eight aforementioned ML methods and compare their performance in order to select the best classification model suitable for our data. To evaluate the eight ML methods’ performance, we apply bootstrap sampling and cross validation [63]. In order to generate reliable results, we use 100 bootstrapping samples for the bootstrap sampling and choose K = 10 for the K-fold cross validation.

Except for LR, all the other models rely on specific assumptions or tuning parameters, which need to be selected in advance. For example, for NB model we need to assume a distribution or generate nonparametric models for the explanatory variables from the training set. We are required to specify the kernel and their hyper parameters for the SVM. For ENET and LAR we need to select the penalty parameters in the loss functions. MARS model requires to specify the pruning method. The tuning parameters for BRT include the loss function (distribution), the tree complexity, the K interaction depth (K-folds), the learning rate parameter *λ* (shrinkage) and the subsampling rate (bag.fraction). In the case of RF, hyper parameters include the number of decision trees in the forest and the number of features considered by each tree when splitting a node.

We use accuracy and Cohen’s Kappa to compare model performance in prediction. Models with higher accuracy and Cohen’s Kappa value are believed to have better performance in prediction and fitness. We select the best one among the aforementioned eight methods as our classification method. From the selected model, we identify the relatively important risk factors for VTE as those variables with p-value less than α (α = 0.05).

**Fig 1:**
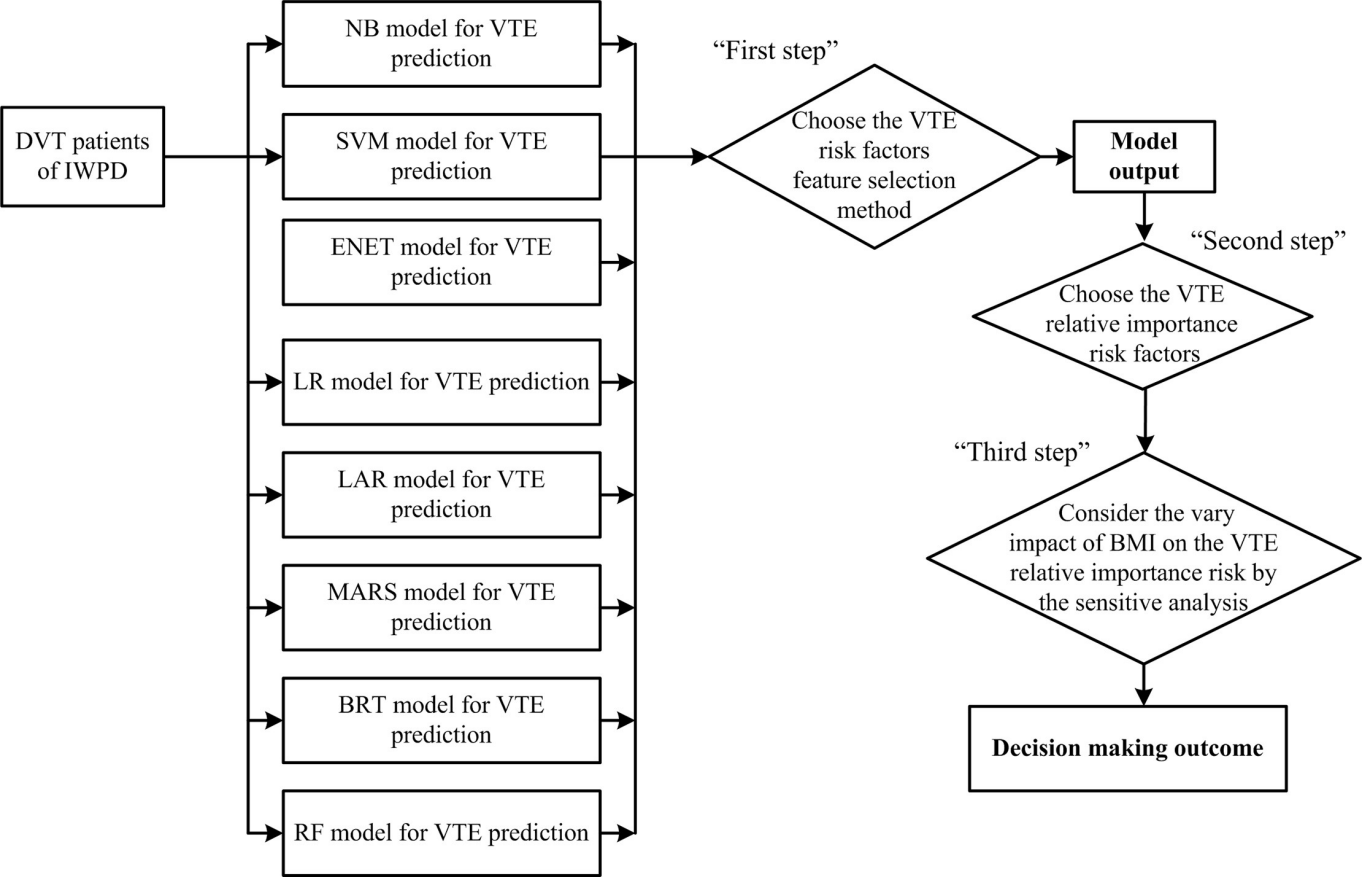
Modelling procedure to evaluate the effect of BMI on VTE risk factors for warfarin users.

The flowchart presented in Fig 1 shows the study workflow. We perform all calculations using R (V3.6.1). In particular we use R packages including “e1071”, “glmnet”, “dismo”, “earth”, “gbm”, “vcrpart”, “caret” and “naivebayes”.

## 3 Data

In this study, we use the International Warfarin Pharmacogenetics Consortium (IWPC) data obtained from the PharmGKB (http://www.pharmgkb.org), to identify important risk factors for VTE and to predict its incidence among DVT patients. The IWPC include information for 6256 warfarin treated patients from 22 research groups over nine countries in four continents [64]. The dataset contains patients’ non-genetic and genetic information such as their clinical characteristics, personalized medications, warfarin therapeutic doses, and genotypes. In particular, two very important genotypes, i.e. CYP2C9 and VKORC1, are available in the dataset, which are significantly related to warfarin therapy [65–68]. The IWPC has been used in several recent studies to model the warfarin dose with individual patient’s clinical characteristics and genetic information [64, 69–73].

In this study, we choose 376 DVT patients as the cohort validation group to predict VTE incidence (262 out of the 376 DVT patients have VTE). These DVT patients use warfarin on daily basis. Therefore in this study we take their warfarin dose, INR and the genotypes of CYP2C9 and VKORC1 as potential risk factors to examine. We also examine other risk factors including demographic factors, BMI and clinical characteristics. Except that the variables of INR and warfarin dose are continuous, all the other risk factors considered in this study are categorical variables. Table 1 presents the demographic features used in this study, including age, gender, race, and genotypes (CYP2C9 and VKORC1), as well as warfarin dose, INR and BMI, and the corresponding frequency and variable names for each feature.

The clinical characteristics considered in this study include the DVT patients’ comorbidities and concomitant medications.

**Table 1:** Demographic characteristics of the DVT validation group.

Demographic characterize	Data characters	Validation group frequency	Variable Name	Demographic characterize	Data characters	Validation group frequency	Variable Name
**Gender**	Male	189	gen	**race**	Black	125	bl
**Age**	> 40yr.	330	age		White	181	wh
**Warfarin dose-mg/wk**	Median	30	wds		Han Chinese	21	Han
	range	7.00–95.00		**CYP2C9 genotype**	*1/*1	282	g11
**INR**	Median	2.5	INR		*1/*2	45	g12
	range	1.5–3.7			*1/*3	24	g13
**BMI**	Median	30	BMI_A		*1/*5	1	g15
	range	14.9–68			*2/*2	3	g22
	Underweight & normal weight	68			*2/*3	7	g23
	overweight	120	BMI_B		*3/*3	1	g33
	Obesity	137	BMI_C	**VKORC1 genotype**	G/G	168	GG
	Morbidly obesity	51	BMI_D		A/G	99	AG
					A/A	31	AA

In Table 2, we present the frequencies for the comorbidities and concomitant medications we examine, as well as their corresponding variable names in the dataset. All the comorbidities and concomitant medications we examine in this study happen to more than 5% of the DVT patients in this dataset.

**Table 2:** Clinical characteristics of the DVT validation group.

Comorbidities & Concomitant drugs	No (%)	Variable names	Comorbidities & Concomitant drugs	No (%)	Variable names
**Abnormal heart rhythm**	35 (9)	aHR	**Diabetes**	88 (23)	dib
**Aspirin**	80 (21)	asp	**History of stroke**	39 (10)	st
**Atorvastatin**	31 (8)	ato	**Hyperlipidemia**	38 (10)	hyL
**Atrial fibrillation**	24 (6)	AF	**Hypertension**	162 (43)	hyT
**Cancer**	46 (12)	can	**Myocardial infarction**	41(11)	infr
**Depression**	28 (7)	dep	**Simvastatin**	64 (17)	Sim

**Fig 2:**
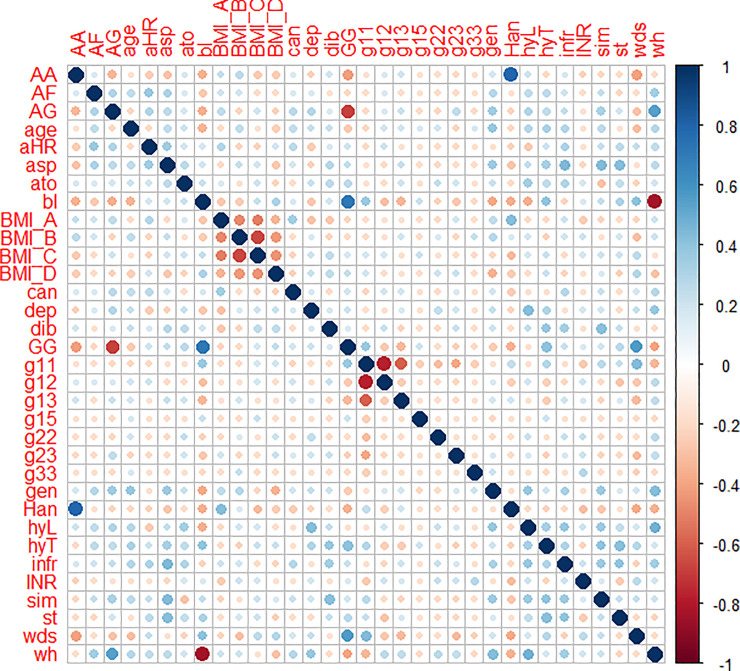
Visualisation of correlations acrosss the risk factors.

We check the correlations across all variables in the DVT patient dataset. Fig 2 presents the correlation vizualization. It is worthwhile to note that the labels in Fig 2 are the variable names listed in Tables 1 and 2, which are in the alphabetical order from left to right and top to bottom. It is common to see high negative correlations across different categories of the same feature, for example “bl” (the race of black) and “wh” (the race of white). Across different features, we see high positive correlations between “Han” and “AA”, and between “bl” and “GG”. It has been found that AA genotype of VKORC1 is the most common genotype in Asians, and the most common genotype in African Americans is GG [74], which can explain the high correlations across the VKORC1 and race categories in our data.

## 4 Results and discussion

As shown in Fig 1, to evaluate the interaction impact of BMI on the other risk factors for VTE, we conduct a three-steps analysis. First, we select the best classification model from the eight ML methods presented in Section 2; second, we identify the important risk factors for VTE using the selected model; third, we examine the interaction impact of BMI on the other risk factors for VTE.

### 4.1 Selection of the best classification model

**Fig 3:**
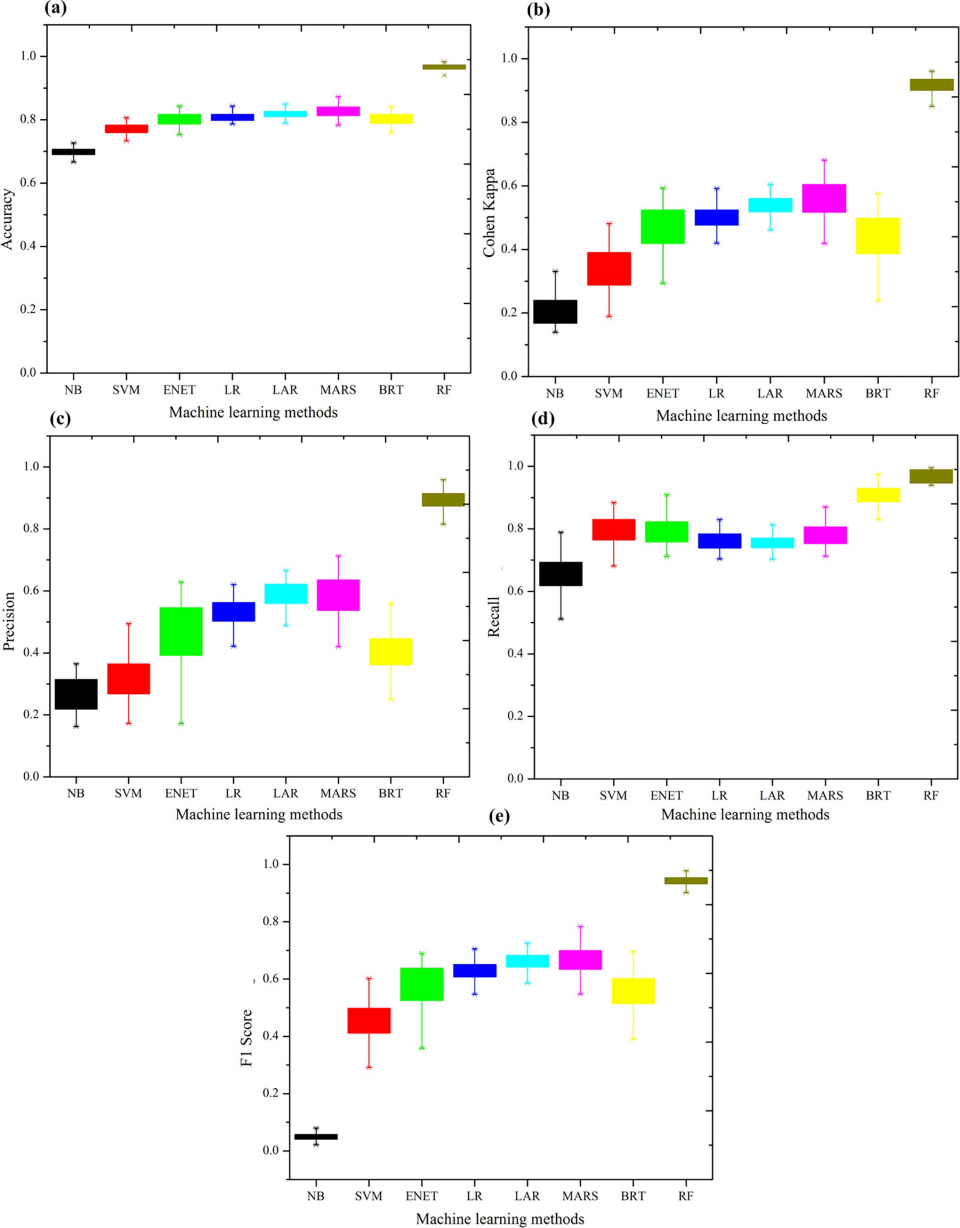
Model performance on prediction.

First of all, we apply the eight ML methods introduced in Section 2, in order to select the best classification method among them. As discussed in Section 2.9, we have to select the required hyper parameter(s) for each ML method except for the LR model. In this study, we use Gaussian distribution for the NB method. For the SVM model, we choose a linear kernel and set the regularization hyper parameter C as 10. In LAR and ENET models, the penalty parameters are selected through cross-validation. We apply the backward strategy as the pruning method for the MARS model. As for the BRT model, Gaussian distribution is chosen as the loss function, while the tree complexity, the learning rate (*λ*) and the subsampling rate (bag.fraction) are set as 4, 0.004 and 0.5, respectively. For the RF model we set the number of trees as 500 and the number of features considered by each tree (mtry) as 4. To evaluate the prediction performance of these ML models, we apply five measures, including accuracy, Cohen’s Kappa, precision, recall and F1 score. For all performance measures, a higher value indicates a better performance on prediction. We perform a bootstrap sampling with replication number of 100 and K-fold (K = 10) cross validation to calculate these measures. The results are presented in Fig 3.

As shown in Fig 3, the RF model has the best prediction performance among the eight ML models, followed the MARS model, and the NB model has the poorest performance. Therefore we choose RF as the best model to conduct the following analysis.

**Fig 4:**
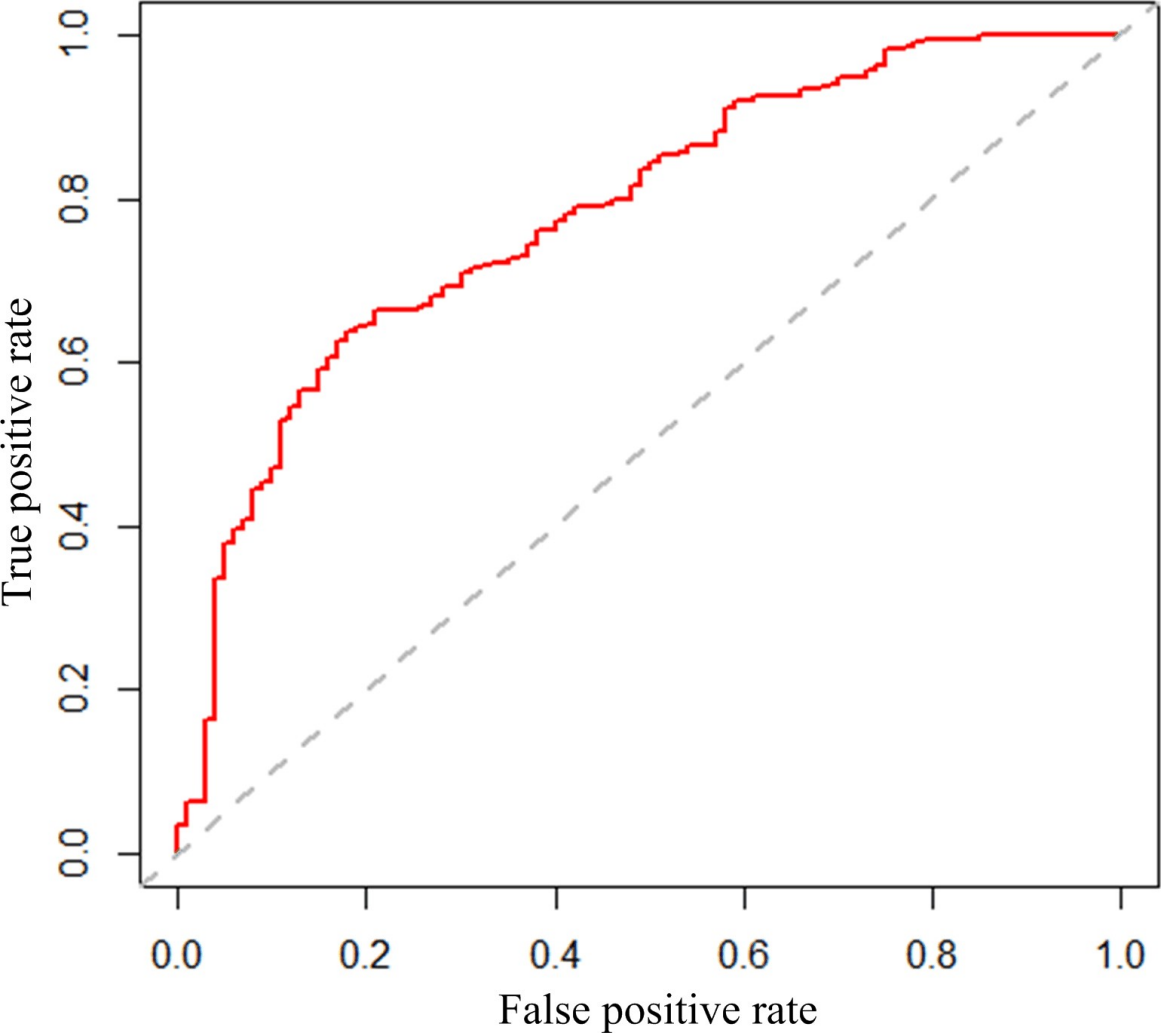
ROC curve of random forest model (RF).

In Fig 4, we plot the Receiver Operator Characteristic (ROC) curve for the RF model. Its AUC is 0.78207, indicating a good performance on classification.

**Fig 5:**
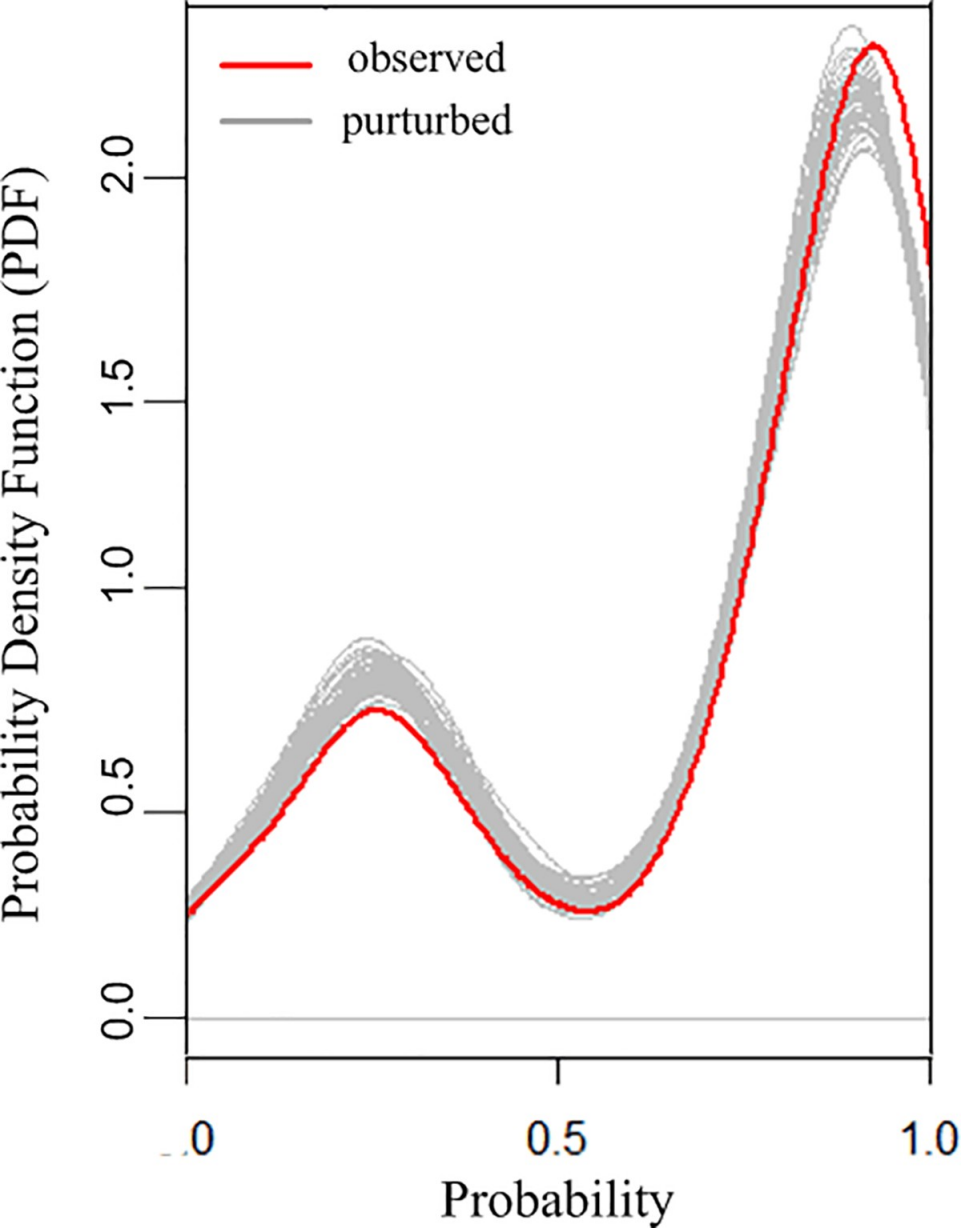
Monte Carlo simulation for model stability.

Next, we implement a simple Monte Carlo simulation to examine the stability of our prediction using the RF model. In this Monte Carlo simulation, we generate a simulation envelope through a number of randomization (for example, n = 50) of VTE risk factors. From each randomization, the probability density function (PDF) of the predicted probability of VTE incidence can be obtained. All these PDFs constitute the simulation envelope. We compare the PDF of the predicted probability of VTE incidence from our dataset with the simulation envelope to examine the stability of the RF model prediction. Fig 5 presents the results on the PDF of the predicted probability of VTE incidence from our dataset (indicated by the red line) and the simulation envelope (indicated by the grey lines).

As shown in Fig 5, the prediction from the RF model (observed) matches well with the simulation envelope (perturbed). In particular, if we define the perturbed error as the difference between the prediction from the RF model and the simulation probability, the error mean is 0.1012 and the standard deviation is 0.0064, which demonstrate the stability of the RF prediction.

### 4.2 Identification of the important risk factors

We apply the RF model to rank importance of risk factors for VTE. Table 3 presents the mean decrease accuracy (MDA) for all risk factors used in this study and the corresponding p-values from permutation tests. The variables with larger mean decrease accuracy values are more important for VTE incidence, and the permutation p-values indicate the significance of importance metrics for the RFmodel.

**Table 3:** MDA of risk factors and permutation p-value.

variables	MDA	P-value	variables	MDA	P-value	variables	MDA	P-value
**Han**	10.7470	0.0196**	**dep**	2.6900	0.0568*	**sim**	0.2702	0.5156
**hyL**	7.1637	0.0215**	**ato**	2.2515	0.1058	**g15**	0.1379	0.5647
**GG**	6.4284	0.0196**	**AG**	2.0170	0.1294	**g22**	-0.0232	0.7215
**wh**	5.6208	0.0215**	**can**	1.7153	0.1470	**g33**	-0.1104	0.6196
**INR**	5.2390	0.0215**	**hyT**	1.5271	0.1921	**BMI_B**	**-0.2345**	**0.8431**
**bl**	4.9470	0.0196**	**g11**	1.2509	0.2372	**BMI_C**	**-0.4384**	**0.6627**
**AF**	4.4381	0.0254**	**wds**	0.9681	0.2862	**age**	-0.6928	0.7921
**aHR**	4.1946	0.0235**	**asp**	0.8348	0.2921	**g13**	-1.0160	0.7411
**AA**	3.6397	0.0313**	**BMI_A**	**0.7155**	**0.3098**	**g23**	-1.3614	0.7960
**infr**	3.3170	0.0607*	**g12**	0.5582	0.4215	**dib**	-1.7603	0.8666
**st**	3.0067	0.0568*	**gen**	0.4266	0.4431	**BMI_D**	**-2.4002**	**0.9470**

We identify the risk factors with high MDA (>2) and low permutation p-values (<0.05) as relatively important risk factors for VTE. In particular, they include race feature (Han, bl, wh), hyperlipidemia (hyL), VKORC1 genotype (GG and AA), INR, atrial fibrilation (AF), abnormal hear beat (aHR), myocardial infarction (infr), stroke (st) and depression (dep).

### 4.3 Impact of BMI

Even though our results from the RF model show BMI is not an important risk factor for VTE, previous studies have found that obesity may interact with other risk factors in VTE development and change the impacts of other risk factors on VTE [22]. However there is very limited evidence on the exact interaction between BMI and the other VTE risk factors. This study aims to fill the gap in the literature. To examine the interaction effects, first we group our sample into four BMI categories, including underweight and normal weight (BMI<25), overweight (25≤BMI<30), obesity (30≤BMI<40), and morbidly obesity (BMI≥40). We then apply the RF model to each BMI group separately. In this way, we can obtain the varying impacts of the other important risk factors, including comorbidities and demographic features, on VTE occurrence for individuals from different BMI groups.

**Fig 6:**
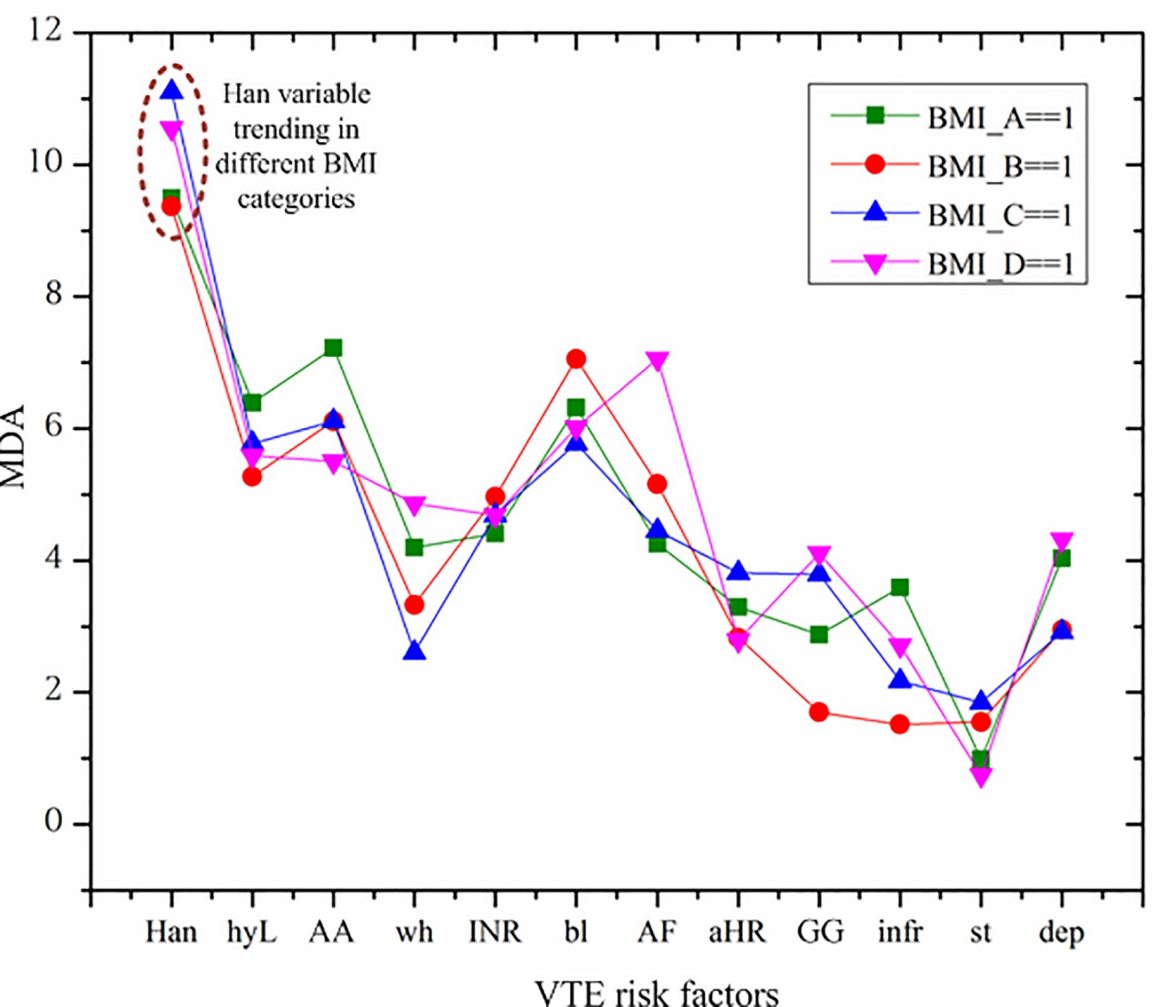
Importance of VTE risk factors in different BMI categories. BMI_A = = 1 for underweight & normal weight, BMI_B = = 1 for overweight, BMI_C = = 1 for obesity, BMI_D = = 1 for morbidly obesity.

Table 4 presents the MDA of the important risk factors (identified by the RF model for the whole sample) for each BMI group. We also present the significance level of permutation test for importance for each risk factor across the BMI groups. As shown in Table 4, all important risk factors for VTE, except for two features (infarction and stroke), are still significantly important for VTE across all different BMI groups. However, infarction and stroke are significantly important for some specific BMI groups. For example, infarction is a moderate and weak VTE risk factor for morbidly obese people and overweight people, respectively, with significance level for importance at 10%, but it is not significantly important for people for the other two BMI groups. Stoke is significantly important for overweight and obese people but not for normal or under weight people, in terms of VTE occurrence. It is worthwhile to mention that the importance of AA genotype for VKORC1 is only 10% significant for the morbidly obesity people, while for all other people, it is 5% significantly important. On the other hand, depression is 5% significantly important for overweight people, but only 10% significantly important for the other BMI groups. In terms of MDA values, all risk factors, except for three feastures including AA genotype for VKORC1, infarction and stroke, can be qualified as, at least, moderate (i.e.MDA>2) risk factors for VTE. In particular, AA genotype for VKORC1 and infarction are two weak VTE risk factors (MDA<2) for overweight individuals but moderate for people from other BMI groups; while stroke is a weak risk factor for any BMI group. It can be seen that importance of the same risk factors are different across different BMI groups. We present the visualized results of MDA across different BMI groups in Fig 6.

**Table 4:** Importance of VTE risk factors across four BMI categories.

VTE relative importance risk factors	MDA			
	Underweight and nornal weight (BMI_A = = 1)	Overweight (BMI_B = = 1)	Obesity (BMI_C = = 1)	Morbidly Overweight (BMI_D = = 1)
**Han**	9.4954**	9.3687**	11.1069**	10.55530**
**hyL**	6.3908**	5.2757**	5.7726**	5.59147**
**GG**	7.2202**	6.1136**	6.1143**	5.50135**
**wh**	4.1953**	3.3283**	2.6041**	4.8617**
**INR**	4.4106**	4.9653**	4.69031**	4.6838**
**bl**	6.32087**	7.05594**	5.7726**	6.0119**
**AF**	4.25188**	5.1580**	4.4514**	7.0565**
**aHR**	3.2959**	2.8318**	3.8149**	2.79094**
**AA**	2.87470**	1.6976**	3.7920**	4.11092*
**infr**	3.59218	1.5147*	2.1701	2.70934*
**st**	0.98655	1.5530*	1.84809*	0.74331*
**dep**	4.03310*	2.9494**	2.9211*	4.31530*

** and * indicate 5% and 10% significant in the permutation test, respectively.

The horizontal axis of Fig 6 indicates the important VTE risk factors identified from the whole sample, which are ordered according to their MDA from highest to lowest. For example, “Han” has the highest MDA of 10.7470 and the lowest MDA of 2.6900 belongs to “dep”, as presented in Table 3. Fig 6 presents four lines of MDA, estimated for the four BMI groups, respectively. The trend of these lines is not strictly downward and these lines are not parallel.

Two conclusions emerge from Fig 6. First, the importance order of these risk factors for a particular BMI group is different from that of the whole population. For example, although the variable of “Han” is the most important VTE risk factor for DVT patients regardless BMI category, the second most important risk factor is different for each BMI group from the whole population. In particular, without taking into account of the interaction effect of BMI, hyperlipidemia is the second most important risk factor, however it is “bl” for overweight and morbidly obesity group, and “GG” for obesity group. Similar differences can be found on the other risk factors.

Second, the same VTE risk factor may play a different role across different BMI groups. Take the variable of race feature, “Han”, as an example, though it is the most important one across all BMI categories, it plays a most important role in the obesity group, followed by morbidly obesity group, and a least important role in the overweight group (as indicated by the points in the dashed oval in Fig 6). For some risk factors, such as “AF”, we can see more variations in the importance across different BMI grooups. But for some variables, such variation is much smaller, such as “INR”. These results further demonstrate the interaction effect of BMI on the other risk factors on VTE incidence.

## 5 Conclusions

In this study, we investigate the interaction impact of BMI on the other important VTE risk factors. First, we apply eight ML methods, including NB, SVM, ENET, LR, LAR, MARS, BRT and RF. According to five performance measures, i.e. accuracy, Cohan’s Kappa, precision, recall and F1-score, we choose the RF model as the best classification model among the eight ML methods. Second, by applying the RF model we identify twelve important risk factors according to their MDA and permutation test for importance. Last, we run the RF model separately for each BMI group to examine the interaction impact of BMI on the other important VTE risk factors. From this three-step analysis, we conclude that, first the importance of VTE risk factors may vary for different BMI groups. For example, we find that the AA genotype of VKORC1 plays a more important role in determine VTE occurrence for obesity or morbidly obesity individuals than for people from the other BMI categories. Second, the variation of a risk factor’s importance on VTE incidence across the four BMI groups is different. For example, we see large variation in the importance of atrial fibrillation across BMI groups, however the variation in INR’s importance is much smaller. Therefore in order to determine the risk of VTE and how risk factors impact on VTE, the interaction impact of BMI on the risk factors has to be taken into account.

## Supporting information

S1 Data (XLS)

## Abbreviations

BFs: basis functions
BRT: boosted regression tree
DVT: deep venous thrombosis
ENET: elastic net regression
LR: logistic regression
INR: international normalized ratio
IWPC: international warfarin pharmacogenetics Consortium
LAR: lasso regression
MARS: multivariate adaptive regression splines
MDA: mean decrease accurency
ML: machine learning
OLS: ordinary least squares
PE: pulmonary embolism
RF: random forest classification
VTE: venous thromboembolism
